# Human-derived IgG level as an indicator for EBV-associated lymphoma model in Hu-PBL/SCID chimeras

**DOI:** 10.1186/1743-422X-8-213

**Published:** 2011-05-09

**Authors:** Yunlian Tang, Rongfang He, Yang Zhang, Fang Liu, Ailan Cheng, Yimou Wu, Runliang Gan

**Affiliations:** 1 Cancer Research Institute, University of South China, Hengyang City, Hunan 421001, P. R. China; 2 Department of Pathology, The First Affiliated Hospital, University of South China, Hengyang 421001, Hunan Province, China

## Abstract

**Background:**

Epstein-Barr virus (EBV) has a close association with various types of human lymphomas. Animal models are essential to elucidate the pathogenesis of human EBV-associated lymphomas. The aim of the present study is to evaluate the association between human IgG concentration and EBV-associated lymphoma development in huPBL/SCID mice.

**Methods:**

Human peripheral blood lymphocytes (hu-PBL) from EBV-seropositive donors were inoculated intraperitoneally into SCID mouse. Immunohistochemical staining was used to examine differentiated antigens of tumor cells. EBV infection of the induced tumors was detected by *in situ *hybridization. IgG concentrations in the serums of 12 SCID mice were measured by unidirectional immunodiffusion assay.

**Results:**

21 out of 29 mice developed tumors in their body. Immunohistochemical staining showed that all induced tumors were LCA (leukocyte common antigen) positive, B-cell markers (CD20, CD79a) positive, and T-cell markers (both CD3 and CD45RO) negative. The tumors can be diagnosed as human B-cell lymphomas by these morphological and immunohistochemical features. In situ hybridization exhibited resultant tumor cells had EBV encoded small RNA-1 (EBER-1). Human-derived IgG could be found in the serum from SCID mice on the 15^th ^day following hu-PBL transplantation, and IgG levels increased with the tumor development in 6 hu-PBL/SCID chimeras.

**Conclusions:**

Intraperitoneal transfer of hu-PBLs from EBV+ donors to SCID mice leads to high human IgG levels in mouse serum and B cell lymphomas. Our findings suggest that increasing levels of human-derived IgG in peripheral blood from hu-PBL/SCID mice could be used to monitor EBV-related human B-cell lymphoma development in experimental animals.

## Background

Epstein-Barr virus (EBV) is a ubiquitous human herpes virus that persists in most human bodies as a lifelong latent infection in host lymphocytes after a primary viral encounter, and it has been confirmed to be the etiological factor of infectious mononucleosis [1,2]. More important, EBV, which may be one of human tumor viruses [3], has a close association with human lymphoma and nasopharyngeal carcinoma [4-6]. Although EBV can transform human lymphocytes and squamous epithelia in vitro, it is impossible to conduct controllable investigation on human body. It is also a difficult problem to induce neoplasm with EBV in animal body. Up to date, no study about infection and oncogenicity of EBV has been done with an ideal animal model.

Animal models of lymphoma are essential to elucidate the pathogenesis of human EBV-associated lymphomas. Severe combined immunodeficient (SCID) mouse (homozygous C.B.-17 scid/scid) expresses a truncated form of the catalytic subunit of the DNA-dependent protein kinase and is unable to properly rearrange the Ig and TCR genes. The ensuing severe combined immunodeficiency endows these mice with the capacity to accept xenografts. Because SCID mice lack functional T or B lymphocytes, they can be engrafted with functioning human hematolymphoid cells to create human/SCID chimeras [7,8]. In immunosuppressed individuals, such as post-transplant patients, the presence of EBV-infected B cells may lead to lymphoproliferative disease [9]. Injection of human peripheral blood lymphocytes (hu-PBLs) or hematopoietic stem cells from EBV-positive donors into SCID mice induces human lymphoproliferative disease in the humanized SCID recipients [10,11]. This xenochimeric human-mouse model can be used to elucidate the mechanisms of EBV-specific lymphomagenesis and to assess novel therapeutic approaches. The aim of the present study is to detect molecular biomarkers of the EBV-induced lymphomas in hu-PBL/SCID mice and to measure serum IgG levels in hu-PBL/SCID chimeras.

## Materials and methods

### Construction of hu-PBL/SCID chimeras

SCID(C.B.-17scid/scid) mice were bought from Laboratory Animal Center of Science Academy in China, 6 to 8 weeks old, 18 ± 2.43 g in weight, male or female. All mice were bred in micro-isolator cages in a specific pathogen-free (SPF) environment. Animal studies were approved by Institutional Animal Care and Use Committee (IACUC) of Chinese Academy of Sciences.

Fresh peripheral venous blood was collected from 12 healthy adult donors by 300 ml per one, and PBLs were separated from heparinized peripheral blood by isopycnic centrifugation on Ficoll-Hypaque. The EBV immune status of donors was assessed by using a standard ELISA for the presence of a serum IgA anti-EBV-viral-capsid-antigen(IgA/VCA). Hu-PBLs from EBV-seropositive donors were inoculated intraperitoneally into 29 SCID mice by 1 × 10^8 ^PBLs resuspended in 1 ml RPMI-1640 medium per mouse, such mice are hereafter referred to as hu/SCID chimeras.

### Assay for human IgG of mouse serum

12 mice were bled from a tail vein on days 3, 7, 15, 22, 33, and 46 post hu-PBLs transplantation, 10-20 μl blood for each mouse every time; serum samples were stored at -80°C until use. The concentrations of human IgG in mouse serums were assessed by unidirectional immunodiffusion assay. Briefly, serum samples were diluted (1:5), and loaded 10 μl/well on the mouse anti-human IgG immunodiffusion plate. The plate was incubated in a wet box at 37°C for 24 h. Then the plate was taken out to have the diameter of the precipitation loop measured. The values of IgG were calculated with the diameter-content contrast table prepared by the manufacturer.

### Animal observation, anatomical and histopathological examination

SCID mice were bred in cages separately, with the abnormities of weight, behavior, fur, abdomen and respiration monitored. If animals were sick, they would be killed and exposed to excess-ethyl-ether, then autopsied. In other SCID mice, follow-up was extended to 135 days. The position of tumor formation and thorax-celiac viscera were under particular observation.

Examining the thorax -celiac cavity, mediastinum and other main organs of mice, such as heart, lung, brain, liver, spleen, kidney, lymphnodes, and etc. Observing the size, shape, color, section and invasion of tumor with naked eye. Each tumor mass was divided into 2 samples: one was immediately frozen at -80°C for DNA analysis; the other part was fixed in 10% neutralized formalin, embedded in paraffin and sectioned at 4 μm for histopathology observation and immunohistochemical examination.

### Immunohistochemical staining of the induced tumor

Tumors were fixed in 10% neutral formalin, embedded in paraffin and cut into 4 μm sections for further study. Immunohistochemical stains were performed to assess differentiated antigens of tumor cells, a panel of monoclonal antibodies against lymphocytes (LCA, CD45), B cells (L26/CD20, CD79a) and T cells (CD45RO, CD3) (purchased from Maxin Co., San Francisco, LA) respectively according to the manufacturer's protocol. Briefly, endogenous peroxidase was quenched with 0.3% hydrogen peroxide. Before the application of the primary antibody, nonspecific binding was blocked with normal non-immune serum, and tissue slides were incubated with each diluted antibody at 4°C overnight. Bound antibody was detected with biotin-conjugated secondary antibody followed by streptavidin-peroxidase and diaminobenzidine (DAB) color reagent. The slides were counter-stained with hematoxylin. A case was regarded as positive if more than 5% of the malignant cells were stained with the antibody, although in practice most positive cases had 20% or more of their cells marked.

### Human-specific Alu detection by PCR

DNA was extracted from tumor tissues as described in protocol. *Alu *PCR was conducted under the following conditions: 95°C predenaturation for 5 min, and then 30 cycles of denaturation at 94°C for 1 min, annealing at 57°C for 1 min and extension at 72°C for 1 min. Primers (sense: 5'-CAC CTG TAA TCC CAG CAG TTT-3', anti-sense: 5'-CGC GAT CTC GGC TCA CTG CA-3') were used to amplify a 221 bp sequence for human-specific *Alu*.

### In situ hybridization of EBV infection

The synthetic oligonucleotide EBER1 probe (sequence: 5'-CTC CTC CCT AGC AAA ACC CTC AGG ACG GCG-3') [10] was end-labeled with digoxigenin by tailing with terminal transferase. The labeling reaction was set up according to the manufacturer's protocol (Boehringer Mannheim Co., Ingelheim, Germany). In situ hybridization using EBER1 probe was carried out as follows. Briefly, the glass slides were pretreated with 2% APES. 4-μm tissue slices of the induced tumors were heated at 70°C for 1 h, and deparaffinaged in xylene, then digested with 0.5% μg/ml proteinase K at 37°C for 10 min, and washed in water. The labeled probes were diluted to a concentration of 100 ng/ml in hybridization medium [25% deionized formamide, 4 × SSC (sodium citrate, sodium chloride), 50 mmol/L NaH_2_PO_4_/Na_2_HPO_4, _1 mmol/L EDTA, 5 × Denhart's, 1 mg/ml ssDNA], and then spotted on to the tissue slices, which were covered with a coverslip. Both DNA probe and target RNA in tissue slices were simultaneously denatured at 70°C for 8 min. Then the sections were hybridized at 37°C for 4 h or overnight. The non-specific or unbound probes were removed by two post-hybridization washes as follows: 2 × SSC, 0.1 × SSC, each for 10 min at room temperature. The slices were blocked with 2% normal goat serum at room temperature for 20 min, incubated with anti-Digoxigenin antibody at 37°C for 30 min, and then washed 3 times. NBT/NCIP was used as the chromogen. Counter-staining was done to enhance visualization with nuclear fast red.

## Results

**Figure 1:**
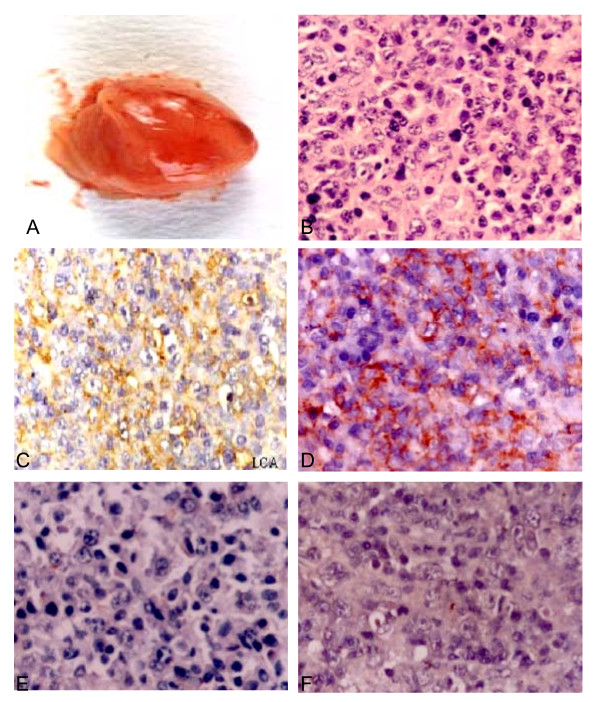
**Morphology and immunological marker examination of EBV-associated tumors in hu-PBL/SCID mice**. (A) Fresh tumor tissue out of SCID mouse showed gray-white and gray-red node, size 17 × 11 × 8 mm. (B) The histopathological type of the induced-tumors was diffuse large cell lymphoma (HE × 400). (C) Positive staining of LCA located on the membrane of the tumor cells (SP × 400). (D) Immunohistochemistry revealed that positive staining of B-cell marker (CD20, L26) located on the membrane of tumor cells (SP × 400). (E) Negative staining of T-cell marker (CD45RO) of the tumor cells (SP × 400). (F) Negative staining of T-cell marker (CD3) of the tumor cells (SP × 400).

The EBV-related neoplasms in the SCID mice were solid masses. 21 out of 29 mice (72%) developed tumors in their body. By autopsy, some were round-shaped nodes (Figure 1A), with distinct boundary and no envelope; others were irregular nodes, adherent to contiguous organs. The diameters of tumors were about 0.5-3.6 cm, and the cuts displayed pale and gray-red. Moreover, loci of necrosis could be observed in the center of larger tumors. Under microscope, tumor cells diffusely arranged with rare sparse interstitial fibers and tiny vessels. The tumors were dominated by large cleaved and non-cleaved cells (Figure 1B), with a few large and diverse immunoblasts and plasmacytoid lymphocytes and a few megalocytes as well. Karyokinesis could be easily observed and nucleoli were distinct in the tumor cells. The tumors in the abdominal cavity of mice often invaded into kidney, liver, pancreas, mesentery; and those in the midiastinum invaded into striated-muscles of posterior thoracic wall. All of the pathological characters revealed that the induced-tumor was highly malignant non-Hodgkin's lymphoma (NHL).

**Figure 2:**
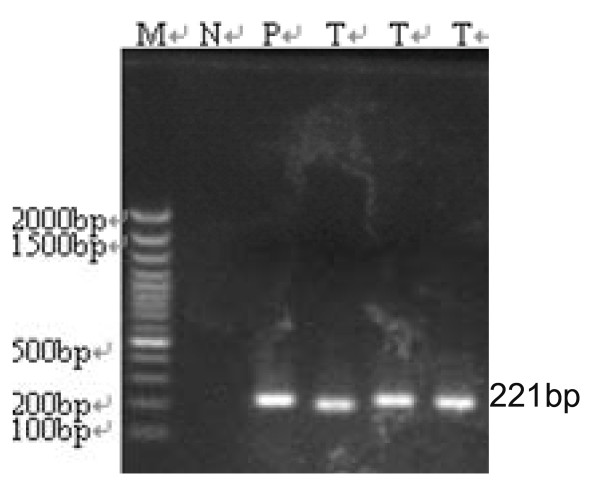
**PCR amplified 221 bp products of human-specific Alu sequence of the EBV-induced tumors in SCID mice**. M: DNA marker, P: human peripheral blood lymphocytes as positive control, N: murine liver tissue as negative control, T: tumor biopsy tissues.

Immunohistochemical staining showed that all induced tumors were LCA (leukocyte common antigen) positive (Figure 1C), B-cell marker (CD20, CD79a) positive (Figure 1D), and T-cell markers (both CD3 and CD45RO) negative (Figure 1E, F). By these morphological and immunohistochemical features, those tumors can be diagnosed as human B-cell lymphomas. Furthermore, *Alu*-PCR showed that all of tumor tissues contained 221 bp *Alu *sequence (Figure 2). It confirmed that the induced tumors in hu-PBL/SCID mice were derived from human, but not from mouse.

**Figure 3:**
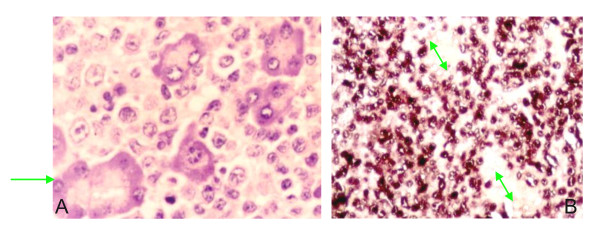
**Detection of EBER in the induced lymphoma of SCID mice**. (A) Diffuse large cell lymphomas invaded into pancreatic tissue (arrow) of SCID mouse by microscopic observation (HE stain × 400). (B) Positive signals of EBER-1 were located in the nuclei of all tumor cells by in situ hybridization, whereas pancreatic epithelial cells (arrow) of SCID mouse were negative (×400).

In biopsy tissue, localization of EBER transcripts by *in situ *hybridization remains the gold standard for identifying latent infection of EBV. The *in situ *hybridization signal of EBER1 probe was very intense with a diffuse pattern of staining confined to the nucleus (sparing the nucleus) in all cases (Figure 3). Notably, almost all morphologically malignant cells exhibited a positive signal with the EBER1 probe, while the normal host tissue adjacent to tumor cells showed negative.

**Figure 4:**
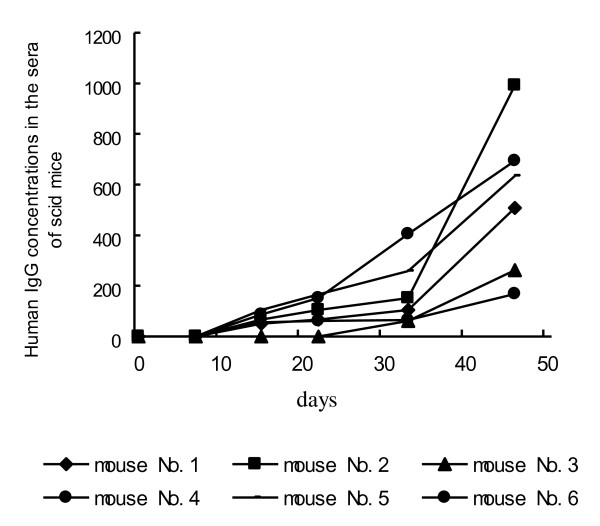
**Human IgG levels in the serums of 6 tumor-bearing mice at different time points**. The concentration of human IgG in the serum of each of SCID mice increased gradually on days 7, 15, 22, 33 and 46 after hu-PBL transplantation.

In the 12 mice whose IgG was examined, human serum IgG could be found in 6 SCID mice on day 15 after the engraftment of hu-PBLs and the IgG concentrations increased with the prolongation of the experiment (Figure 4), and then visible tumors developed. Marked elevations of human serum IgG levels in hu-PBL/SCID mice are associated with EBV-related lymphoma development. Human serum IgG levels from hu-PBL/SCID mice can be considered as a useful index of prediction for oncogenesis and tumor development. In contrast, IgG could not be detected both in the 3 mice with micro-lymphoproliferative lesions only and in the other 3 mice with no tumors during the experimental course. Additionally, our data showed human IgG concentrations in the serum of each of hu-PBL/SCID chimeras were also positively related to the serum IgG levels from different donors.

## Discussion

With the development of human organ transplantation and the increase of AIDS cases in recent years, both lymphoproliferative disease (LPD) in the immunocompromised patients and its relationship with EBV have become increasingly followed with interest [1]. Clinically, almost all post-transplantation lymphomas are associated with EBV infection [12,13]. Post-transplant lymphoprolifertive disorder (PT-LPD) is a syndrome of uncontrolled lymphoid growth in the immunosuppressed transplant patient. In the patients with PT-LPD, the incidence varies from 0.5% after bone marrow transplantation to 12% after heart-lung transplantation. PT-LPD remains so far a complication with a high morbidity and mortality. EBV is associated with posttransplant lymphoproliferative disease (PTLD), which is a leading cause of cancer death in recipients of transplants. On the other hand, investigations for AIDS-related lymphomas show that about 50-80% of those tumors are concerned with EBV [14]. The vast majority of AIDS-NHL belongs to three kinds of high-grade B-cell lymphomas: Burkitt's lymphoma, immunoblastic lymphoma, and large-cell lymphoma [15]. Although there are some differences between this immunodeficiency-associated lymphoproliferative disorders, they share several common features: a tendency to present in extranodal sites, rapid clinical progression when untreated, diffuse large cell histology, B-cell origin and association with the EBV [16]. The occurrence of lymphoma needs the activation, proliferation and transform of B-cell, as well as the development of malignant phenotype. The present experiment showed that B-cell lymphoma in the hu-PBL/SCID chimeras was similar to EBV-related neoplasms in the immunocompromised patients, so it could further indicate that immunodeficiency of animal or human body is an essential condition for the tumorigenesis.

EBV is a herpesvirus associated with approximately 1% of tumours worldwide. EBV is the epitome of B lymphotropic viruses, but the spectrum of tumours associated with EBV extends to various types of human malignancies and carcinomas [1]. Ubiquitous EBV infection in humans implies that most individuals carry EBV-infected cells. Therefore, mere detection of the virus in individuals with a tumour is not sufficient for establishing a causal relationship between both events. Currently there is no in vivo model that can adequately recapitulate EBV infection and its association with B-cell lymphomas. In this experiment, we can observe the development of EBV-related lymphoma in the SCID mice engrafted with hu-PBLs from EBV-seropositive donors. 72% of mice (21/29) developed tumors in EBV-positive hu-PBL/SCID chimeras. The tumors possess solid, aggressive and fatal features, and belong to high malignant NHL in histopathology. Both Alu-gene detection and immune marker examination showed that the induced-tumors were derived from human B-cell, and the tumor cells contained EBV-encoded small RNA (EBER). We have successfully induced human B-cell lymphomas in the body of hu-PBL/SCID chimeras. An in vivo model of human CD20+ B-lymphoma was established in SCID mice with huPBLs transplantation from EBV-seropositive donors.

Our present experiment showed that intraperitoneal transfer of hu-PBLs from EBV+ donors to SCID mice could generate human-derived B cell lymphomas. But it is necessary for us to monitor EBV-associated human B-cell lymphoma development in the body of SCID mice. IgG is an immunoglobulin secreted by plasmocyte when mature B cells are differentiated into plasmocytes by antigen stimulation. It is reported that human-derived IgG can be used to assess whether immune function of SCID mouse with hu-PBL engraftment is reconstructed [17,18] and that antigen stimulation promotes human secondary IgG responses in hu-PBL/SCID mouse [19,20]. Interestingly, a significantly positive relationship exists between the appearance of human IgG and tumorigenesis of B-cell lymphoma in the findings of our present study. Those SCID mice in which human serum IgG continuously increased would finally developed visible tumors in their bodies. In the mice without human serum IgG during all the experimental phases, no tumor or only micro-lymphaproliferative lesions was identified with microscope. From our data, human serum IgG concentrations in hu-PBL/SCID chimeras were also related to the serum IgG levels from original donors. However, the IgG level from a donor was not related to the development of tumors. It meant that all B-lymphocytes that produce different IgG levels from various donors had the same potential to develop lymphomas if activated by EBV. Moreover, it is of importance and practice for us to replicate the tumor model in the future. So we can predict the tumorigenesis by detecting human serum IgG levels from hu-PBL/SCID mice during the experimental phases. More importantly, it can give us a dependable serum marker if we want to test therapeutic intervention on the EBV-induced lymphomas.

## Abbreviations

EBV: Epstein-Barr virus; hu-PBL: human peripheral blood lymphocyte; SCID: severe combined immunodeficiency;

## Competing interests

The authors declare that they have no competing interests.

## Authors' contributions

YT and RH carried out construction of hu-PBL/SCID chimeras, participated in the animal observation, anatomical and histopathological examination, drafted and revised the manuscript. YZ, FL and AC participated in the immunoassays and in situ hybridization. YW and RG participated in the design of the study and revised the manuscript. All authors read and approved the final manuscript.
